# Metastatic Tumors from Unknown Primary in Cervical Lymph Nodes: A Decade of Study in a Tertiary Care Center of a Resource-Limited Country

**DOI:** 10.31729/jnma.v64i293.9292

**Published:** 2026-01-31

**Authors:** Shankar Kafle

**Affiliations:** 1 Maharajgunj Medical Campus/Tribhuvan University Teaching Hospital, Department of Pathology, Maharajgunj, Kathmandu, Nepal

**Keywords:** *cervical region*, *lymph node*, *malignancy*, *metastasis*, *primary*

## Abstract

**Introduction:**

Cervical lymphadenopathy may be the first clinical manifestation of an occult primary tumor, and it is a crucial area of investigation due to its significance in cancer diagnosis and prognosis. This study aimed to establish the spectrum of malignancies metastasizing to cervical lymph nodes of unknown primary origin and to analyze their distribution by age, sex, and nodal site.

**Methods:**

A retrospective descriptive study was conducted over a decade (2015 - 2024 AD) in Tribhuvan University Teaching Hospital (TUTH). All the cases of metastatic tumors in cervical lymph nodes with unknown primary origin were included.

**Results:**

A total of 174 cases were studied. Age of patient ranges from 5 years to 90 years, with a mean age of 58±17.3 years. There were 12 (6.89%) lung cancer and 9 (5.17%) thyroid cancer as primary site. On histological examination, there were 44 (25.28%) adenocarcinoma and 25 (14.36%) squamous cell carcinoma.

**Conclusions:**

Lung cancer was more common followed by thyroid and adenocarcinoma was the most common histological findings. The supraclavicular lymph node was most often involved in this study.

## INTRODUCTION

There are multiple sites and several lymph nodes in the cervical region^1^ that might be the first clinical manifestation of an occult primary tumor. Almost 1% of all head and neck malignancies are due to metastases from a remote primary site.^2^ This can be the first clinical manifestation of an occult primary tumor in 2-5% of cases.^3^ Some of them may present as airway emergencies.^4^ Different types of cancers exhibit characteristic histopathological features when they metastasize to cervical lymph nodes.^5^ Histopathology, with or without immunohistochemistry is crucial for cancer diagnosis and prognosis assessment.^6^ In cases of unidentified sites of primary tumor, most centers advocate treatment including wide-field radiotherapy, which results in significant morbidity.^7^ The aim of this study was to find the spectrum of malignancies that metastasize to cervical lymph nodes.

## METHODS

This is a descriptive study conducted with retrospective data from 2015 to 2024. All records of cervical lymphadenopathy who were diagnosed as metastatic tumors during the study period was included in the study. Cervical lymphadenopathy was defined as nodes over 1 cm in length during examination of head and neck region.^8^ Cases were retrieved from the medical records and database of Department of Pathology, Tribhuvan University Teaching Hospital (TUTH) following ethical approval from Institutional Review Committee (Reference number: 79/081/082). Only those cases diagnosed as metastatic malignancy involving the cervical lymph nodes without a defined primary site were included, and cases of lymphoma were excluded. The duplicate case, as well as repeated samples from different cervical lymph nodes, were also excluded. Similarly, cases with no proper data and histopathological slides to review were also excluded. All available records, along with histopathological diagnoses, including the slides, were reviewed and reconfirmed. Data was collected manually using a proforma and entered in Microsoft Excel (version 2019), and analysis was conducted. Histopathological and clinical data were retrieved from medical records and pathology archives and were reviewed systematically. Relevant clinical history and diagnostic findings were extracted and analyzed. Cervical lymph node level were classified as per their primary drainage site (Table 1).

**Table 1:** Cervical lymph nodes with their primary drainage site.^1^

Cervical Lymph Node Level	Primary drainage site
Level I(CX I)	Anterior oral cavities, lip, and sinonasal region
Level II(CX II)	Posterior oral cavity, oropharynx, supraglottic larynx, and parotid gland
Level III(CX III)	Larynx (glottic and subglottic) and hypopharynx
Level IV(CX IV)	Subglottis, thyroid, and cervical esophagus
Level V(CX V)	Nasopharyngeal and skin over the occipital scalp and neck
Level VI(CX VI)	Pharynx, thyroid, subglottis, and cervical esophagus
Level VII(CX VII)	Thyroid, subglottis, and cervical esophagus

**Table 2:** Primary site of malignancy (organ specific) that metastasize in lymph nodes in cervical regions (n=174).

Primary site of malignancy (organ-specific) that metastasizes to lymph nodes in the cervical region	n(%)
Lung	12(6.89)
Thyroid	9(5.17)
Ovary	3(1.72)
Breast	1(0.57)
Gastrointestinal tract	1(0.57)
Not with a suggestive primary site	148(85.05)

## RESULTS

**Figure 1:**
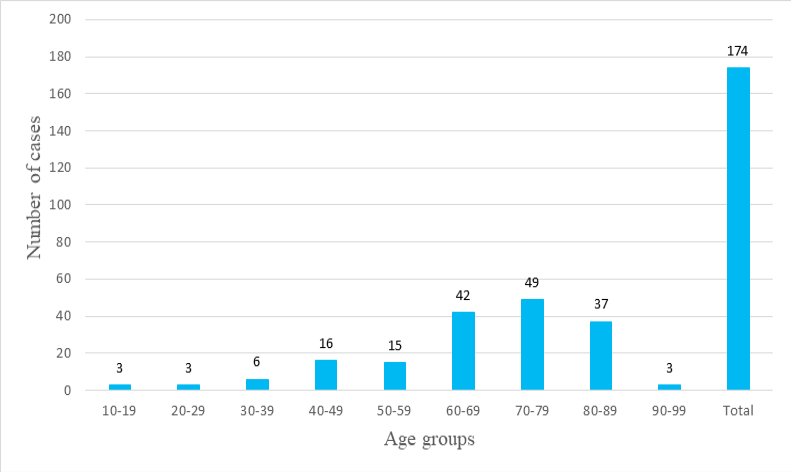
Age distribution of patients with metastatic tumors from unknown primary in cervical lymph nodes (n=174).

During the period of 10 years, there were a total of 174 cases of cervical lymphadenopathy with metastatic tumor of unknown primary site were identified. Male to female ration was 1.4:1. The mean age of patient was 58±17.3 years with age ranging from 5 to 90 years. There were 49 (28.16%) patients in age group 70-79 years (Figure 1).

There were 12 (6.89%) lung cancer and 9 (5.17%) thyroid cancer as primary site (Table 2).

**Figure 2:**
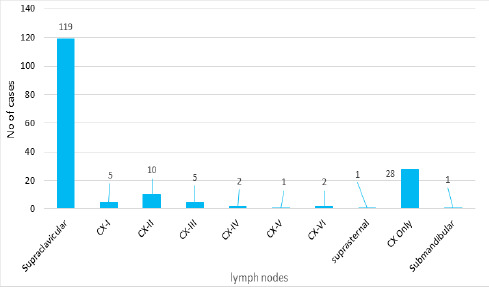
The trend of lymph node involvement (CX=Cervical) (n=174).

Histopathologically, there were 44 (25.28%) adenocarcinoma, 25 (14.36%) squamous cell carcinoma, 11 (6.32%) poorly- differentiated carcinoma, 8 (4.59%) small cell carcinoma, 6 (3.44%) papillary thyroid carcinoma and 1 (0.57%) of medullary carcinoma, nasopharyngeal carcinoma, and small round cell tumor each. There were 119 (68.39%) cases of supraclavicular lymph nodes and 28 (16.09%) cervical lymph node involvement(Figure 2). Trends of cases per year rose in an increasing pattern from 3 in 2015 to 47 in 2024 AD.

## DISCUSSION

Different types of cancers may exhibit characteristic histopathological features when they metastasize to cervical lymph nodes. Such lesions may be of various malignancies. Squamous cell carcinoma, adenocarcinoma, melanoma, lymphomas, undifferentiated carcinomas, neuroendocrine carcinomas, papillary thyroid carcinomas, and many more rare types of malignancies. For example, cervical metastasis of squamous cell carcinoma from an unknown primary site occurs in 2% to 4% of head and neck tumors.^5^ Diagnosing malignant cervical lymphadenopathy is still challenging.^9^ Therefore, in this study, a good deal of effort was spent attempting to establish a comprehensive spectrum of diverse types of malignancies that metastasize in cervical lymph nodes, as well as to identify occult primary tumor sites in the patient group.

Among 174 cases studied, the commonest primary site was diagnosed as lung 12 (6.89%), followed by thyroid 9 (5.17%). This was like a study done by Ellison E. et al., in which most malignancies originated in the lung, breast, or cervix. The left or right side did not discriminate for either the presence or type of tumor.^10^ According to a study by Lopez F et al., the commonest site of remote primary to neck lymph nodes was breast, which was only after the lung, thyroid, and ovary in this study.^2^ However, they stated that the chances of breast carcinoma metastasizing to the neck were low, i.e. <5%.

About the supraclavicular lymph nodes, these are a paired group of lymph nodes located on each side in the hollow superior to the clavicle, close to the sternoclavicular joint. It is the final common pathway of the lymphatic system as it joins the central venous system. These lymph nodes transport lymph from the thoracic cavity and abdomen. The right supraclavicular lymph node is the drainage of the mid-section of the chest, esophagus, and lungs. One of the left supraclavicular lymph nodes, known as the Virchow node, drains the thoracic duct, abdomen, and thorax. It is adjacent to the junction where incoming lymph is introduced back into the venous circulation through the left subclavian vein. Some malignancies, such as lung, head and neck, breast, esophageal, gastric, pancreatic, gynecologic, and prostate cancers, have a propensity to metastasize to supraclavicular lymph nodes. A few clues in clinical examinations can also be present to identify the nodal metastasis, for example, the Troisier sign is the name given to left-sided supraclavicular lymphadenopathy, highly suggestive of abdominal malignancy.^11^

Capodiferro S et al., stated that the supraclavicular group was the most often involved when neck metastases occurred from lung, although the involvement of level I and II nodes had also been reported.^12^ A similar result was in our study, in which supraclavicular involvement was in 11 cases out of 12, and the remaining case was cervical node. In a study by Asimakopoulos P et al., cervical node metastasis was common in papillary thyroid carcinomas, with an incidence ranging between 40%-60%.^13^ which was like this study, of which 88.89% were papillary thyroid carcinomas and 11.11% were medullary carcinoma of the thyroid. The cervical II group was most often involved, followed by the supraclavicular, although other sites were CX III, IV, V, VI.

As per Sivanandan R et al., patients in the anterolateral group (levels II, III, and IV) were at the greatest risk of metastatic disease, with level III nodes consistently the most frequently involved, across all treatment groups. In their study, only three patients exhibited level I involvement, all of whom had extensive neck disease involving all or almost all neck levels.^14^. In this study, Level I involvement was in 5 cases, and all were on the right side, although it was not the comment lymph node involvement.

In a study done on 228 cases by Baqher Hussaini SA et al., the commonest site of metastasis of breast carcinoma was axillary lymph nodes (33.77%), followed by cervical (22.50%) and pelvic (9.64%).^15^ Our study was not concordant with that study, as only one case with supraclavicular (right) was present, and results may vary with an increase in the number of cases. According to Kim JS et al., supraclavicular lymph node (SCL) metastasis was revised from stage IV to stage IIIC in 2003 by the International Union Against Cancer/American Joint Committee on Cancer (AJCC) staging system, as the survival outcomes of patients were found like those with stage IIIB disease and significantly better than those of patients with visceral stage IV.^16^

In this study, the age of patients ranged from 5 years to 90 years with a mean age of 58 years, showing a broad range of age distribution. The commonest age group was 70-79 (28.16%). This was like numerous studies done in the past, for example, studies by Capodiferro S. et al., and Baqher Hussaini SA et al.^11,15^. In our case, the youngest age was 5 years. The case was a case of rhabdomyosarcoma metastasized in the cervical lymph node, and another six-year-old male child was also there with neuroblastoma in the supraclavicular lymph node.

When evaluated with a light microscope, various morphologies can differentiate the specific type of tumor.^7^ According to Varadhachary GR et al., adenocarcinoma was the commonest type in ~60%, followed by poorly differentiated adenocarcinoma or undifferentiated carcinoma or neoplasm (~30%-35%), and squamous cell carcinoma (~5%) or neuroendocrine cancers (~2%). Other types, which were occasionally presented as mixed tumors, were adenosquamous carcinoma, adenocarcinoma with neuroendocrine components, or sarcomatoid carcinoma.^7^ In this study, adenocarcinoma was the commonest type of malignancy 44 (25.28%) followed by squamous cell carcinoma 25 (14.36%). Poorly differentiated carcinoma was next to the squamous cell carcinoma. This was also similar to the study by Pavlidis, N. et al., which showed almost half of the cases had adenocarcinoma.^17^ In our study, poorly differentiated carcinoma was present in 11 (6.32%) cases. Others were 8 (4.59%) cases of small cell carcinoma, 6 (3.44%) cases of papillary thyroid carcinoma, and a single case of medullary carcinoma, nasopharyngeal carcinoma, and small round cell tumor. However, a greater percentage 77 (44.25%) were metastatic carcinoma. This might be due to a tiny tissue bit or a scant amount of tumor cells to define the type of tumor.

The commonest lymph node involved was supraclavicular 119 (68.39%) followed by Cervical level II (CX-II) 10 (5.74%), as shown in Figure 2. In a study done by Keerthana KM, Level II: nodes were the most involved nodes in head and neck cancer.^1^ Our study revealed lungs were the commonest site 12 (6.89%), and the commonest lymph node metastasis was supraclavicular, which was supported by the study done by Capodiferro S et al.^11^

Trends of cases per year rose in an increasing pattern from 3 to 47 cases in a decade. Such an increase might be due to the increasing incidence of malignancy or cancer awareness and improved diagnostic facilities. However, it should be taken into consideration as a concern for further study and needs to be included in national policy and plans to control the burden of malignancies in our country.

Cervical lymph node metastasis, manifested as the first clinical manifestation of an occult primary tumor, could be diagnosed by histopathology and immunohistochemistry, where significant in cancer diagnosis and prognosis.^4^ This study focused on the evaluation of cervical lymphadenopathy for metastatic tumors, so it helped in early diagnosis and localization of the primary site of the tumor for proper management of malignancy. In the previous decades, with the arrival of new imaging technologies and biochemical markers, it was believed that the role of surgery in the diagnosis of cervical lymphadenopathy would be decreased. However, biopsy is still the gold standard for the diagnosis of cervical lymphadenopathy. ^9^

While previous studies had explored the prevalence of infectious pathology in cervical lymph nodes and determined tuberculosis as the commonest cause.^17^ The scenario changed; cervical lymphadenopathy should be evaluated for the metastatic tumor.^2^

Extensive work-up with specific pathology investigations (immunohistochemistry, electron microscopy, molecular diagnosis) and modem imaging technology [computed tomography (CT), mammography, Positron Emission Tomography (PET) scan] resulted in some improvements in diagnosis; however, the primary site of some cases could remain unknown, even on autopsy.^18^

One of the primary limitations of our study was the challenge in obtaining histopathological slides due to logistical constraints or damage. However, almost all the data was saved in computerized medical records. Similarly, we relied on existing data from medical records or databases, which resulted in a small number of patients, given the rarity of the condition. A small number of samples might affect the result, as accuracy increases with an increase in sample size of a study.

## CONCLUSION

The medical presentation of cervical lymphadenopathy frequently masquerades as different pathological conditions, which might be manifested as the first clinical manifestation of an occult primary tumor. Lung cancer was more common followed by thyroid and adenocarcinoma was the most common histological findings. The supraclavicular lymph node was most often involved in this study.

## Data Availability

The data are available from the corresponding author upon reasonable request.
